# Parasite accumulation in placenta of non-immune baboons during *Plasmodium knowlesi* infection

**DOI:** 10.1186/s12936-015-0631-5

**Published:** 2015-03-18

**Authors:** Faith I Onditi, Onkoba W Nyamongo, Charles O Omwandho, Naomi W Maina, Fredrick Maloba, Idle O Farah, Christopher L King, Julie M Moore, Hastings S Ozwara

**Affiliations:** Department of Tropical and Infectious Diseases, Institute of Primate Research, PO Box 24481-00502, Karen, Nairobi Kenya; Department of Biochemistry, University of Nairobi, PO Box 30197-00100, Nairobi, Kenya; Department of Biochemistry, Jomo Kenyatta University of Agriculture and Technology, PO Box 62000-00200, Nairobi, Kenya; Center for Global Health and Disease, Case Western Reserve University, Wolstein Research Building 4-132, 2103 Cornell Road, Cleveland, OH 44106 USA; Department of Infectious Diseases, College of Veterinary Medicine, University of Georgia Athens, Athens, GA 30602-7387 USA

**Keywords:** Baboons, Non-immune, Placental malaria, *Plasmodium knowlesi*, Accumulation

## Abstract

**Background:**

Placental malaria (PM) causes adverse pregnancy outcomes in the mother and her foetus. It is difficult to study PM directly in humans due to ethical challenges. This study set out to bridge this gap by determining the outcome of PM in non-immune baboons in order to develop a non-human primate model for the disease.

**Methods:**

Ten pregnant baboons were acquired late in their third trimester (day 150) and randomly grouped as seven infected and three non-infected. Another group of four nulligravidae (non-pregnant) infected was also included in the analysis of clinical outcome. Malaria infection was intravenously initiated by *Plasmodium knowlesi* blood-stage parasites through the femoral vein on 160^th^ day of gestation (for pregnant baboons). Peripheral smear, placental smear, haematological samples, and histological samples were collected during the study period. Median values of clinical and haematological changes were analysed using Kruskal-Wallis and Dunn’s Multiple Comparison Test. Parasitaemia profiles were analysed using Mann Whitney U test. A Spearman’s rank correlation was run to determine the relationship between the different variables of severity scores. Probability values of P <0.05 were considered significant.

**Results:**

Levels of white blood cells increased significantly in pregnant infected (34%) than in nulligravidae infected baboons (8%). Placental parasitaemia levels was on average 19-fold higher than peripheral parasitaemia in the same animal. Infiltration of parasitized erythrocytes and inflammatory cells were also observed in baboon placenta. Malaria parasite score increased with increase in total placental damage score (r_s_ = 0.7650, P <0.05) and inflammatory score (r_s_ = 0.8590, P <0.05). Although the sample size was small, absence of parasitized erythrocytes in cord blood and foetal placental region suggested lack of congenital malaria in non-immune baboons.

**Conclusion:**

This study has demonstrated accumulation of parasitized red blood cells and infiltration of inflammatory cells in the placental intravillous space (IVS) of baboons that are non-immune to malaria. This is a key feature of placental falciparum malaria in humans. This presents the baboon as a new model for the characterization of malaria during pregnancy.

## Background

Every year, 125 million women are exposed to the risk of malaria worldwide [1]. In sub-Saharan Africa where malaria burden is high, *Plasmodium falciparum* causes up to 10,000 cases of malaria-related deaths in pregnancy, mainly due to maternal anaemia, and approximately 200,000 infant deaths annually [2]. In these women, malaria parasites accumulate and sequester in the placental intervillous space (IVS), a condition referred as placental malaria (PM) [3]. PM leads to complications that threaten the lives of both mother and foetus, such as stillbirths, pre-term deliveries, low birth weights, reduction in gestation period, anaemia, and mortality [4,5]. This has socio-economic impact on the affected population [6].

Over the years, substantial efforts have been made to prevent and control malaria by employing different strategies [2,6,7]. These efforts have encountered major challenges, such as lack of effective vaccines, inadequate animal models and rampant drug resistance [2]. Studies on PM immunopathology have been conducted in rodents. The shortcoming of these studies is that data obtained cannot be correlated to humans because of their difference in reproductive system [8-10]. To bridge this gap, animal models such as non-human primates whose reproductive system mimics the human situation are required in order to produce reliable data [11-13]. Baboons are good animal models for malaria in pregnancy because they have a similar host-pathogen interaction and a reproductive system that is physiologically similar to that of humans [14-18]. This study was designed to describe pathological features and clinical outcomes associated with PM in *Plasmodium knowlesi*-infected baboons. Results from this study will contribute to the validation of the baboon-*P. knowlesi* model of malaria in pregnancy.

## Methods

### Experimental animals and parasite isolate

Fourteen female adult baboons (*Papio anubis*) weighing between 12 Kg and 23 Kg were acquired from the animal facility of Institute of Primate Research (IPR), Kenya, and housed in individual squeeze-back cages (dimensions 0.6×0.6×0.68 m) in the biocontainment facility according to institutional standards. The animals were fed on a standard non-human primate diet, water provided *ad libitum* and their general health monitored throughout the experimental period [19]. Prior to use, they were determined to be free from simian immunodeficiency virus (SIV), haemoprotozoan and gastrointestinal parasites. Of the 14 baboons, ten were time-mated for the first time (primigrravidae) and their pregnancies confirmed by ultrasound, while four were utilized as nulligraids (non-pregnant).

### Experimental infections of baboons

Malaria infection was initiated with 2×10^5^*P. knowlesi* H strain, Pk1 (A+) clone blood stage parasites [14,19]. Four out of the ten pregnant baboons and the four nulligravidae were intravenously infected via the femoral vein. For the pregnant baboons whose gestation period is 180 days, infection was initiated on 160^th^ day of their gestation. Prior to infection, cryopreserved *P. knowlesi* parasites were retrieved and cultured overnight by modification of Rowe *et al*. method [15,20]. Parasitaemia levels, clinical changes and haematological profiles were monitored throughout the experimental period. Caesarean section (CS) was performed on day 8 post-infection and on the 169^th^ day of gestation for non-infected control baboons, in order to collect intact placentas. Oral treatment with pyrimethamine (1 mg/kg of body weight for three days) was initiated after CS for the pregnant animals and at peak parasitaemia for nulligravidae.

### Sample collection

Peripheral blood samples were collected for haematological analysis at three intervals, i.e., baseline (before infection), after infection and after treatment, respectively. Intact placentas were harvested during CS according to standard operating procedures and the delivered infants taken to the nursery soon after birth. Placental and umbilical cord blood was collected according to Brustoski *et al*. [21] and Moore *et al*. [22], respectively. Umbilical cord and chorionic membrane was stripped off and the placenta placed in sterile 0.1% heparin and 2% penicillin-streptomycin saline buffer.

### Parasitaemia and clinical observation

In order to assess the progression of the disease, baboons were monitored for parasitaemia and clinical symptoms, such as fever, appetite, lethargy, and weight changes. Peripheral parasitaemia was determined daily by finger-prick method from day 2 post infection. To determine placental and cord parasitaemia, blood samples collected in capillary tubes were used [21,22]. Thin blood smears were prepared for each animal and parasitaemia determined as described by Ozwara *et al.* [14].

### Haematological and pathological analysis

Venous blood was collected in heparin tubes and haemoglobin (Hb), red blood cells (RBC) and white blood cells (WBC) counts determined at baseline, after infection and after treatment. Gross pathological examination was done during CS by a veterinary pathologist. Placentas were observed for any form of extensive tearing, damage or gross abnormalities. Several placental biopsy specimens of approximately 2×2 cm were obtained for each placental sample, fixed in 10% neutral buffered formalin and later stained with haematoxylin-eosin (H&E). The stained tissues were examined according to Davison *et al.* [11] with a few modifications in order to determine the level of placental damage, inflammatory infiltration, parasite infiltration, and pigmentation scores.

### Statistical analysis

Data was analysed using Graph Pad Prism (version 5.0). Experimental animals were grouped as pregnant infected, nulligravidae infected and pregnant not infected. Median values of clinical and haematological changes of these groups were compared using Kruskal-Wallis statistical test and further analysed using Dunn’s multiple comparison test. Parasitaemia profiles were analysed using Mann Whitney U test. A Spearman’s rank correlation was run to determine the relationship between the different variables of severity scores. Probability values of P <0.05 were considered significant.

### Ethical approval

This study was carried out under the Malaria Research Programme at the Institute of Primate Research (IPR) located in Karen, Nairobi, Kenya. The Institutional Review Committee (IRC) comprising animal care and use committee (ACUC), scientific review and the research ethics and integrity team approved all protocols and use of animals in this study.

## Results

### Clinical and haematological outcome

**Figure 1:**
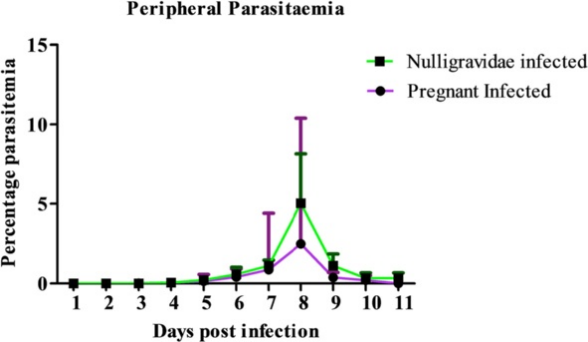
**Comparison of peripheral parasitaemia in pregnant infected and nulligravidae infected baboons.** Nulligravids had approximately 50% higher levels of peripheral parasitaemia compared to pregnant baboons. The difference was not significant (P = 0.4439). Bars show the mean ± SD.

**Figure 2:**
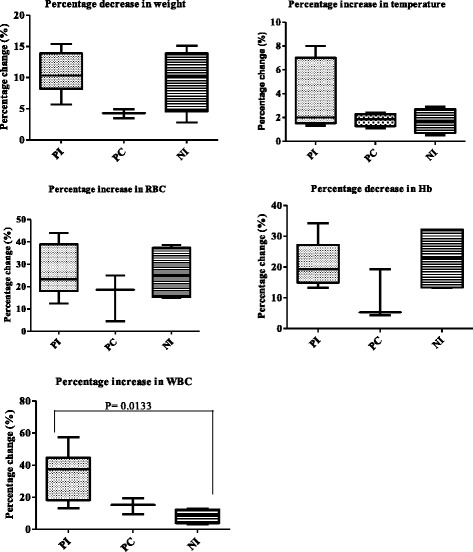
**Clinical and haematological changes in pregnant infected (PI), pregnant control (PC) and nulligravidae infected (NI) before infection (baseline) and after infection.** Increase in WBC was significant (P = 0.0133) between pregnant infected and nulligravidae infected.

In this study, malaria patency was observed on days 2 and 4 post-infection (PI) in nulligravidae and pregnant infected baboons, respectively. Although peak peripheral parasitaemia (at day 8 PI) was on average 50% higher in nulligravids compared to pregnant baboons, this change was not significant (P = 0.4439) (Figure 1). Changes in weight (P = 0.0925), temperature (P = 0.651), red blood cells (RBC) (P = 0.6121), and Hb (P = 0.12379) levels were not significant. Levels of white blood cells (WBC) increased significantly (P = 0.0133). Further analysis by Dunn’s multiple comparison test revealed that increase in WBC levels was significantly higher between pregnant infected and nulligravidae infected (Figure 2).

### Gross pathology in *Plasmodium knowlesi*-infected baboons

The harvested dams were maroon-red in colour with intact membranes. The maternal surface of the placenta had cotyledons while the foetal surface was densely distributed with blood vessels. Baboon placentas weighed on average 167.3 g and 165.9 g for the infected and non-infected groups, respectively. All animals had no fibrinoids except one (pan3233), which had slightly marked fibrinoids. Slight placental calcification was observed in all groups. Cord insertion was intact either centrally or eccentrically placed and with three blood vessels regardless of the group. Placental praevia was complete and no haemorrhage, whether retro-placental or retro-membranous was observed in all the examined placentas. These observed features present similarity of baboon placenta to human placenta.

### Absence of parasitized erythrocytes in cord blood

**Figure 3:**
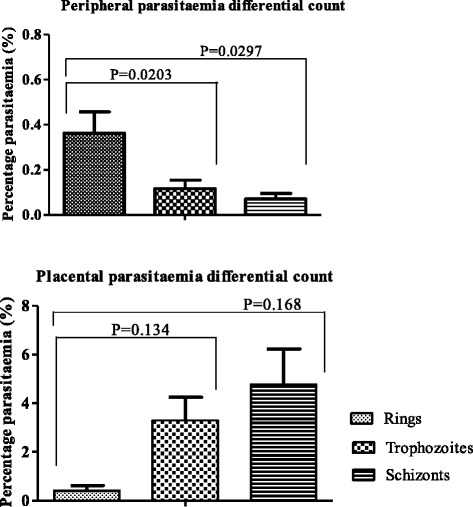
**Parasitaemia differential counts in peripheral and placental blood samples.** Ring stage was significantly dominant in peripheral circulation while mature parasite forms (trophozoites and schizonts) were significantly dominant in placental circulation. Bars show the mean ± SD.

**Figure 4:**
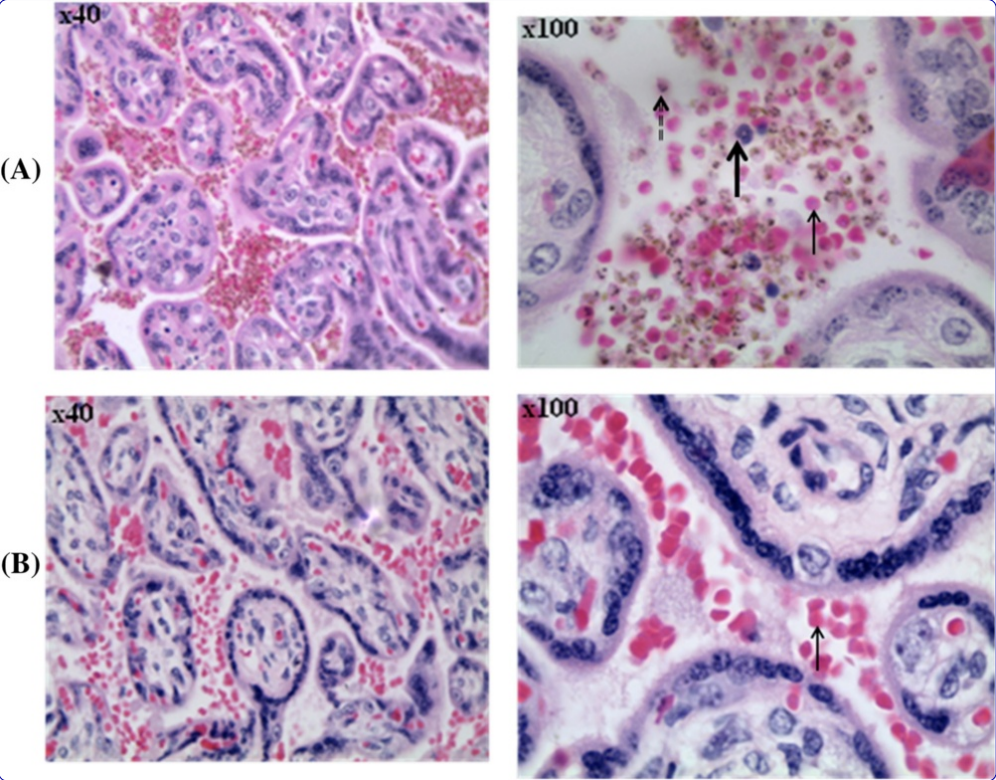
**Photomicrograph showing placental tissue of H&E-stained placental biopsies.** Placental tissue from *P. knowlesi*-infected baboon **(A)** is described by infiltration of parasitized red blood cells (broken arrow), inflammatory cells (thick arrow) and non-parasitized erythrocytes (thin arrow) compared to tissue from non-infected baboon **(B)**. The volume of in filtered red blood cell increases in the baboon placenta following *P. knowlesi* infection.

**Figure 5:**
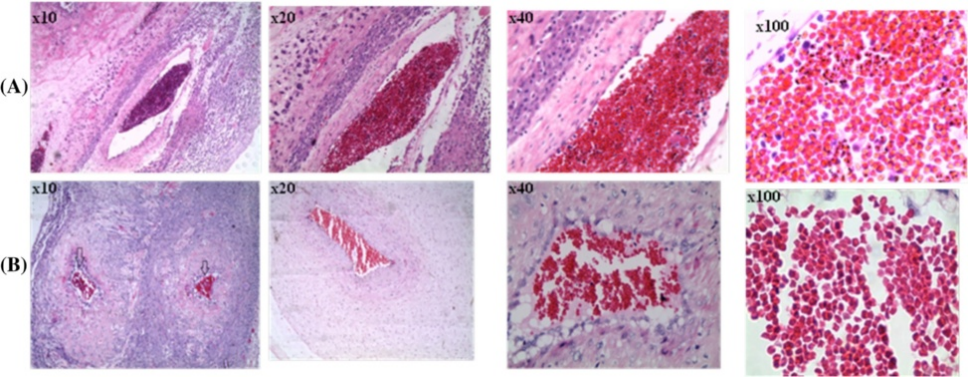
**Photomicrograph representing (A) placental maternal region (basal plate) and (B) placental foetal region (chorionic plate) of H&E-stained placenta of** ***P. knowlesi*** **-infected baboon (Pan 3233).** Parasitized erythrocytes and immune cells are observed in **(A)** the basal plate (maternal side) and absence of parasitized cell in the **(B)** foetal red blood cells on the chorionic plate (foetal side) in slides.

Thin blood smears prepared from placental maternal surface, foetal surface, cord blood, and maternal peripheral circulation were observed and compared for parasitaemia levels. Placental parasitaemia (from maternal region) was on average 19-fold higher than peripheral parasitaemia in the same animal. This difference was significant (P = 0.00823). Parasitaemia differential count demonstrated the abundance of rings and late stage parasites (schizonts) in peripheral and placental (maternal side) blood samples respectively (Figure 3). In spite of high parasitaemia levels in blood smears from placental maternal section, the absence of parasitized RBC in smears from cord blood and placental foetal region was observed (Table 1). This was further confirmed by histology where H&E slides showed absence of parasitized erythrocytes on the foetal vessel and placental chorionic plate (Figures 4 and 5). Infiltration of parasitized erythrocytes and inflammatory cells such as macrophages, neutrophils and monocytes in the intravillous space (IVS) was observed in the baboon placenta (Figure 4).

**Table 1:** **Parasitaemia levels in** ***P. knowlesi*** **infected baboons at caesarean delivery**

**Baboon number**	**Group**	**Peripheral parasitaemia (%)**	**Placental parasitaemia (cotyledon) (%)**	**Placental parasitaemia (chorion) (%)**	**Cord parasitaemia (%)**	*Fold increase in placental parasitaemia
2724	Pregnant	0.80	18.31	-	-	22.89
3305	Pregnant	0.69	4.46	-	-	6.5
2809	Pregnant	0.03	0.74	-	-	24.67
3314	Pregnant	0.92	4.8	-	-	5.2
2859	Pregnant	0.39	14.25	-	-	36.54
3392	Pregnant	0.6	4.3	-	-	7.2
3233	Pregnant	0.58	18.23	-	-	31.43
2870	Nulligravidae	0.8	-	-	-	-
2911	Nulligravidae	4.6	-	-	-	-
3023	Nulligravidae	0.09	-	-	-	-
3035	Nulligravidae	0.5	-	-	-	-

*Fold increase was calculated as placental parasitaemia (from maternal side) over peripheral parasitaemia.

### Placental damage in *Plasmodium knowlesi*-infected baboons

**Figure 6:**
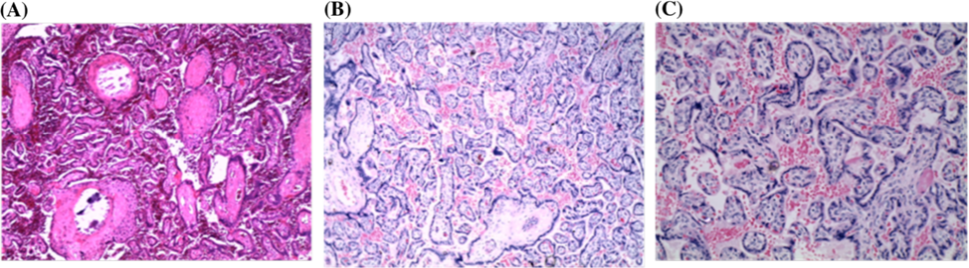
**Photomicrograph of villi showing fibrin necrosis in H&E placental biopsies of (A) infected and (B and C) non-infected baboon at ×10 and ×20 magnifications, respectively.** Necrosis is more severe in malaria-infected placental tissue.

H&E-stained placental biopsies were analysed by severity score ranging from 0 to 4, with 0 representing none, 1 representing minimal and 4 representing the most severe according to Davison *et al.* [11] with a few modifications. The total score for each parameter was recorded on all layers of the placenta and the average obtained. The infected group had significantly higher scores for damage and inflammation compared to the control group (Table 2). Total placental damage score consisted of fibrin necrosis of the villi (Figure 6), chorionic plate thrombosis, syncytiotrophoblast disruption and chorionic plate syncytiotrophoblast disruption. Malaria parasite score increased with increase in total placental damage score (r_s_ = 0.7650, P <0.05) and inflammatory score (r_s_ = 0.8590, P <0.05). This study demonstrated that placental damage and infiltration of immune cells was directly associated with *P. knowlesi* infection in baboons and subsequent accumulation in the placenta.

**Table 2:** **Histopathological scores from H&E stained baboon placental tissues**

**Group**	**Number of animals**	**Mean total placental damage score (TPDS)**	**Mean malaria pigment score (MPS)**	**Mean inflammatory score (IS)**	**Mean parsitaemia score (PS)**
**Pregnant infected**	7	12.00	5.79	4.71	7.29
**Pregnant control**	3	6.67	0.00	1.50	0.00

## Discussion

This study demonstrated, for the first time, the infiltration of parasitized RBC and inflammatory cells in the placentas of non-immune baboons. During *P. knowlesi* infection in pregnancy, symptomatic disease is manifested in combination with increased placental parasitaemia. Consequently, accumulation of parasites in the placental IVS combined with infiltration of inflammatory cells leads to placental damage.

In areas of stable malaria transmission, asymptomatic infections in pregnant women are common, with low clinical malaria presentation due to acquired immunity over time. In contrast, pregnant women in unstable malaria transmission areas, such as the Asia-Pacific region and South America present clinical disease because of low levels of acquired immunity [23]. This study demonstrated higher levels (50%) of parasitaemia in nulligravids compared to pregnant infected baboons, although the difference was not significant. In addition, higher levels of placental parasitaemia were observed compared to peripheral parasitaemia in the same animal. The distribution of parasites in an infected pregnant woman varies with endemicity. The proportion of parasitized erythrocytes is often higher in the placenta than in the peripheral blood in endemic areas [24,25] because infected erythrocytes are preferentially retained in the placenta [26,27]. Haemozoin has also been frequently observed in placentas from malaria-infected women whether in the presence or absence of peripheral parasitaemia [4,28,29]. These findings correlate well with human studies.

Placental falciparum malaria in humans increases neonatal mortality by lowering birth weight while fever is associated with premature birth [30]. Increase in body temperature and reduction in RBC and Hb levels are also observed. According to human studies, it is documented that anaemia is associated with low birth weight, a major determinant of infant mortality associated with *P. falciparum* [30]. It is estimated that approximately 500,000 pregnant women develop severe anaemia due to *falciparum* malaria [31,32] and up to 10,000 maternal anaemia-related deaths are as a result of malaria infection in sub-Saharan Africa [33,34]. Severe anaemia is common in children and pregnant women in regions of stable, high malaria transmission such as sub-Saharan Africa, while mild anaemia is common in semi-immune or non-immune populations [35,36]. Reduced level in Hb during malaria infection is an indicator of anaemia while high parasitaemia density has been implicated for reduced Hb levels [37]. A similar observation was demonstrated in this study as displayed by the reduced Hb and RBC levels in *P. knowlesi*-infected non-immune baboons.

Increased levels of WBC in peripheral circulation and inflammatory cell (monocytes, macrophages and neutrophils) infiltration in H&E-stained tissues obtained from *P. knowlesi-*infected baboon placentas were observed in this study. Immunosuppression during pregnancy is important in maintaining the foetal allograft. Although some murine and human studies have demonstrated that suppression of cell-mediated immune responses plays a major role in the increased susceptibility to malaria during pregnancy [24,25,38-40], a study conducted in a *Plasmodium falciparum* hyperendemic area in Tanzania revealed a marked increase in the levels of monocytes and macrophages and cytotoxic T cells in the IVS of placentas with active malaria infection [41]. Findings in this baboon model indicate that placental malaria is not likely to be associated with cell-mediated immunosuppression. The role of immunosuppression in increasing risk to falciparum malarial infection as observed in pregnant women could not be clearly confirmed in this study.

Retroplacental transmission of falciparum malaria parasites from mother to her foetus is rare in humans particularly in CS deliveries. For instance, a study conducted in Burkina Faso, a region of stable malaria transmission, malaria rate in umbilical cord blood occurred in 1.4% of all newborns. This mechanism of congenital transmission was not clear, although it was attributed to several factors [42,43]. However, no such study has been conducted in areas of low or unstable malaria transmission although documented evidence indicates that malaria parasites affect the placenta selectively and are recognized in maternal erythrocytes. In addition, the placenta at full term presents an adequate barrier that prevents malaria parasites from crossing over to the foetal circulation [41,44]. This is similar to what was observed in this study. Although the sample size was small, the constant absence of parasites in placental foetal region and cord blood implies that congenital malaria in non-immune baboons is rare.

This study has also demonstrated accumulation of parasitized erythrocytes in the IVS of the baboon placenta, in addition to high levels of mature forms of the parasite in the placenta. This suggests possible cytoadherence of *P. knowlesi* parasites in the baboon placenta. It is documented that only the mature falciparum malaria parasites show cytoadherence properties [45]. These parasitized erythrocytes adhere to the endothelium via parasite-derived proteins expressed on their surface [46]. In fact, syncytiotrophoblast cells of the human placenta expresses different and variable amounts of host cell receptors onto which the parasites can bind [45,46]. The principal molecule that mediates adhesion of infected erythrocytes is *P. falciparum* erythrocyte membrane protein 1 (PfEMP-1), a large, highly variant parasite antigen protein, encoded by the *var* multigene family [47]. The adhesion phenotypes are not homologous and as a result different parasites can bind to various numbers and combinations of host molecules, such as chondroitin sulphate A (CSA), hyaluronic acid (HA) and Fc receptors [48-50]. In seeking to identify the ligand and receptor molecules associated with accumulation of *P. knowlesi* infected erythrocytes in the baboon placenta, chondroitin sulphate proteoglycan (CSPG) 4 and HAPLN 1 were predicted as putative receptor molecules in the baboon with high similarity to human CSA and HA respectively. In addition, *P. knowlesi* erythrocyte binding proteins (EBP-*alpha*, EBP-*beta* and EBP-*gamma*) matched closely to the placental *P. falciparum* ligand *Var2csa* [51]. Further work is required to demonstrate the precise ligand-receptor molecules responsible for accumulation of *P. knowlesi* parasites in baboon placenta.

Histopathological findings demonstrated that PM in baboons is characterized by placental damage due to fibrinous necrosis of the villi, chorionic plate thrombosis, syncytiotrophoblast disruption, chorionic plate syncytiotrophoblast disruption, and thickening of trophoblastic basement membrane. The process was also accompanied with infiltration of inflammatory cells in the placental tissue was. These findings correlate well with human studies where placental falciparum malaria pathology is characterized by excess perivillous fibrinoid deposits, excessive syncytial knotting, trophoblastic membrane thickening, which have been associated with destruction/damage of placental tissues, and proliferation of cytotrophoblastic cells [8,10,25,46,52]. It is hypothesized that placental damage, especially the thickening of the trophoblastic basement membrane, alters the maternal foetal exchange, leading to malaria-associated placental lesions and poor foetal outcomes [47]. The same mechanism is likely to take place in *P. knowlesi* associated PM in the baboon.

## Conclusions

Pathological and clinical features of *knowlesi* PM in baboons mimic placental falciparum malaria in humans. Compared to other non-human primates used previously, the *P. knowlesi*-baboon model has demonstrated accumulation of parasitized erythrocytes in the placental IVS, a key feature of placental falciparum malaria in humans. Therefore, this study presents the new baboon model for characterization of malaria in pregnancy. This will, for example, be relevant in understanding host molecular mechanisms involved in sequestration, a key determinant of pathogenesis associated with falciparum and knowlesi malaria in pregnancy.

## Abbreviations

ACUC: Animal care and use committee
CS: Caesarean section
CSA: Chondroitin sulphate A
CSPG4: Chondroitin sulphate proteoglycan 4
EBP: Enhancer binding protein
HA: Hyaluronic acid or hyaluronan
HAPLN1: Hyaluronan and proteoglycan link protein 1
Hb: Haemoglobin
H&E: Haematoxylin and eosin
IPR: Institute of Primate Research
IRC: Institutional Review Committee
IVS: Intravillous space
PfEMP-1: *Plasmodium falciparum* erythrocyte membrane protein-1
PI: Post-infection
PM: Placental malaria
RBC: Red blood cells
SIV: Simian immunodeficiency virus
WBC: White blood cells

## References

[1] Conroy AL, McDonald CR, Kain KC (2012). Malaria in pregnancy: diagnosing infection and identifying fetal risk. Expert Rev Anti Infect Ther.

[2] WHO. World Malaria Report 2013. World Health Organization, Geneva [http://www.who.int/malaria/publications/world_malaria_report_2013/report/en/]

[3] Desai M, ter Kuile FO, Nosten F, McGready R, Asamoa K, Brabin B (2007). Epidemiology and burden of malaria in pregnancy. Lancet Infect Dis.

[4] Menendez C, Ordi J, Ismail MR, Ventura PJ, Aponte JJ, Kahigwa E (2000). The impact of placental malaria on gestational age and birth weight. J Infect Dis.

[5] Steketee RW, Wirima JJ, Slutsker L, Breman JG, Heymann DL (1996). Comparability of treatment groups and risk factors for parasitemia at the first antenatal clinic visit in a study of malaria treatment and prevention in pregnancy in rural Malawi. Am J Trop Med Hyg.

[6] Sachs J, Malaney P (2002). The economic and social burden of malaria. Nature.

[7] WHO. World Malaria Report 2011. World Health Organization, Geneva [http://www.who.int/malaria/world_malaria_report_2011/en/]

[8] Davison BB, Cogswell FB, Baskin GB, Falkenstein KP, Henson EW, Tarantal AF (1998). *Plasmodium coatneyi* in the rhesus monkey (*Macaca mulatta*) as a model of malaria in pregnancy. Am J Trop Med Hyg.

[9] King BF (1993). Development and structure of the placenta and fetal membranes of nonhuman primates. J Exp Zool.

[10] Martin CB, Ramsey EM (1970). Gross anatomy of the placenta of rheusus monkeys. Obstet Gynecol.

[11] Davison BB, Cogswell FB, Baskin GB, Falkenstein KP, Henson EW, Krogstad DJ (2000). Placental changes associated with fetal outcome in the *Plasmodium coatneyi/*rhesus monkey model of malaria in pregnancy. Am J Trop Med Hyg.

[12] Khan-Dawood FS, Kanu EJ, Dawood MY (1993). Baboon corpus luteum: presence of oxytocin receptors. Biol Reprod.

[13] Wilson L, Parsons MT, Flouret G (1991). Forward shift in the initiation of the nocturnal estradiol surge in the pregnant baboon: is this the genesis of labor?. Am J Obstet Gynecol.

[14] Ozwara H, Langermans JAM, Maamun J, Farah IO, Yole DS, Mwenda JM (2003). Experimental infection of the olive baboon (*Paplio anubis*) with *Plasmodium knowlesi*: severe disease accompanied by cerebral involvement. Am J Trop Med Hyg.

[15] Nyawira T, Gicheru M, Kagasi E, Ng’ang’a Z, Ozwara H (2012). Acute *Plasmodium knowlesi* Infection in olive baboons (*Papio anubis*) is accompanied by high-level of gamma interferon. Int J Biol.

[16] Weatherall DJ, Miller LH, Baruch DI, Marsh K, Doumbo OK, Casals-Pascual C (2002). Malaria and the red cell. Hematol Educ Program Am Soc Hematol Am Soc Hematol Educ Program.

[17] Nyachieo A, Kiulia NM, Arimi MM, Chai DC, Mwenda JM (2009). Vaginal histological changes of the baboon during the normal menstrual cycle and pregnancy. East Afr Med J.

[18] Chai D, Cuneo S, Falconer H, Mwenda JM, D’Hooghe T (2007). Olive baboon (*Papio anubis anubis*) as a model for intrauterine research. J Med Primatol.

[19] Olobo JO, Reid GDF (1990). Mitogenic responses of peripheral blood mononuclear cells of vervet monkeys (*Cercopithecus aethiops*): Apparent role of adherent cells. Am J Primatol.

[20] Rowe AK, Rowe SY, Snow RW, Korenromp EL, Schellenberg JRA, Stein C (2006). The burden of malaria mortality among African children in the year 2000. Int J Epidemiol.

[21] Brustoski K, Kramer M, Möller U, Kremsner PG, Luty AJF (2005). Neonatal and maternal immunological responses to conserved epitopes within the DBL-gamma3 chondroitin sulfate A-binding domain of *Plasmodium falciparum* erythrocyte membrane protein 1. Infect Immun.

[22] Moore JM, Nahlen BL, Misore A, Lal AA, Udhayakumar V (1999). Immunity to placental malaria. I. Elevated production of interferon-gamma by placental blood mononuclear cells is associated with protection in an area with high transmission of malaria. J Infect Dis.

[23] Takem EN, D’Alessandro U (2013). Malaria in pregnancy. Mediterr J Hematol Infect Dis.

[24] Bulmer JN, Rasheed FN, Francis N, Morrison L, Greenwood BM (1993). Placental malaria. I. Pathological classification. Histopathology.

[25] Walter PR, Garin Y, Blot P (1982). Placental pathologic changes in malaria. A histologic and ultrastructural study. Am J Pathol.

[26] Barasa M, Gicheru MM, Kagasi AE, Ozwara SH (2010). Characterisation of placental malaria in olive baboons (*Papio anubis*) infected with *Plasmodium knowlesi* H strain. Int J Integr Biol.

[27] Watkinson M, Rushton DI (1983). Plasmodial pigmentation of placenta and outcome of pregnancy in West African mothers. Br Med J (Clin Res Ed).

[28] Bulmer JN, Rasheed FN, Morrison L, Francis N, Greenwood BM (1993). Placental malaria. II. A semi-quantitative investigation of the pathological features. Histopathology.

[29] Galbraith RM, Fox H, Hsi B, Galbraith GM, Bray RS, Faulk WP (1980). The human materno-foetal relationship in malaria. II. Histological, ultrastructural and immunopathological studies of the placenta. Trans R Soc Trop Med Hyg.

[30] Luxemburger C, McGready R, Kham A, Morison L, Cho T, Chongsuphajaisiddhi T (2001). Effects of malaria during pregnancy on infant mortality in an area of low malaria transmission. Am J Epidemiol.

[31] Uneke CJ (2007). Impact of placental *Plasmodium falciparum* malaria on pregnancy and perinatal outcome in sub-Saharan Africa. Yale J Biol Med.

[32] Steketee RW, Nahlen BL, Parise ME, Menendez C (2001). The burden of malaria in pregnancy in malaria-endemic areas. Am J Trop Med Hyg.

[33] Guyatt HL, Snow RW (2001). Malaria in pregnancy as an indirect cause of infant mortality in sub-Saharan Africa. Trans R Soc Trop Med Hyg.

[34] WHO (1992). The prevalence of anaemia in women: a tabulation of available information.

[35] Menendez C, Fleming AF, Alonso PL (2000). Malaria-related anaemia. Parasitol Today.

[36] Newton CR, Warn PA, Winstanley PA, Peshu N, Snow RW, Pasvol G (1997). Severe anaemia in children living in a malaria endemic area of Kenya. Trop Med Int Health.

[37] McElroy PD, ter Kuile FO, Lal AA, Bloland PB, Hawley WA, Oloo AJ (2000). Effect of *Plasmodium falciparum* parasitemia density on hemoglobin concentrations among full-term, normal birth weight children in western Kenya, IV. The Asembo Bay Cohort Project. Am J Trop Med Hyg.

[38] Leopardi O, Naughten W, Salvia L, Colecchia M, Matteelli A, Zucchi A (1996). Malaric placentas. A quantitative study and clinico-pathological correlations. Pathol Res Pract.

[39] Ordi J, Ismail MR, Ventura PJ, Kahigwa E, Hirt R, Cardesa A (1998). Massive chronic intervillositis of the placenta associated with malaria infection. Am J Surg Pathol.

[40] Ismail MR, Ordi J, Menendez C, Ventura PJ, Aponte JJ, Kahigwa E (2000). Placental pathology in malaria: a histological, immunohistochemical, and quantitative study. Hum Pathol.

[41] Ordi J, Menendez C, Ismail MR, Ventura PJ, Palacín A, Kahigwa E (2001). Placental malaria is associated with cell-mediated inflammatory responses with selective absence of natural killer cells. J Infect Dis.

[42] Malhotra I, Mungai P, Muchiri E, Kwiek JJ, Meshnick SR, King CL (2006). Umbilical cord-blood infections with *Plasmodium falciparum* malaria are acquired antenatally in Kenya. J Infect Dis.

[43] Ouédraogo A, Tiono AB, Diarra A, Bougouma ECC, Nébié I, Konaté AT (2012). Transplacental transmission of *Plasmodium falciparum* in a highly malaria endemic area of Burkina Faso. J Trop Med.

[44] Rogerson SJ, Mwapasa V, Meshnick SR (2007). Malaria in pregnancy: linking immunity and pathogenesis to prevention. Am J Trop Med Hyg.

[45] Berendt AR, Ferguson DJ, Newbold CI (1990). Sequestration in *Plasmodium falciparum* malaria: sticky cells and sticky problems. Parasitol Today.

[46] Hviid L, Marinho CRF, Staalsoe T, Penha-Gonçalves C (2010). Of mice and women: rodent models of placental malaria. Trends Parasitol.

[47] Baruch DI (1999). Adhesive receptors on malaria-parasitized red cells. Best Pract Res Clin Haematol.

[48] Beeson JG, Amin N, Kanjala M, Rogerson SJ (2002). Selective accumulation of mature asexual stages of *Plasmodium falciparum*-infected erythrocytes in the placenta. Infect Immun.

[49] Duffy PE (2007). Plasmodium in the placenta: parasites, parity, protection, prevention and possibly preeclampsia. Parasitology.

[50] Muthusamy A, Achur RN, Bhavanandan VP, Fouda GG, Taylor DW, Gowda DC (2004). *Plasmodium falciparum*-infected erythrocytes adhere both in the intervillous space and on the villous surface of human placenta by binding to the low-sulfated chondroitin sulfate proteoglycan receptor. Am J Pathol.

[51] Nyamagiri JO, Onditi FI, Ochola L, Waihenya R, Ozwara HS (2014). *Plasmodium knowlesi* Ligand-receptor Process in Baboon (Papio anubis) Placenta. J Biol Agric Healthc.

[52] Yamada M, Steketee R, Abramowsky C, Kida M, Wirima J, Heymann D (1989). *Plasmodium falciparum* associated placental pathology: a light and electron microscopic and immunohistologic study. Am J Trop Med Hyg.

